# Evaluation of the Healthy Lifestyles Initiative for Improving Community Capacity for Childhood Obesity Prevention

**DOI:** 10.5888/pcd15.170306

**Published:** 2018-02-22

**Authors:** Marcie Berman, Frances Bozsik, Robin P. Shook, Emily Meissen-Sebelius, Deborah Markenson, Shelly Summar, Emily DeWit, Jordan A. Carlson

**Affiliations:** 1 Center for Children’s Healthy Lifestyles and Nutrition, Children’s Mercy Kansas City, Missouri

## Abstract

**Purpose and Objectives:**

Policy, systems, and environmental approaches are recommended for preventing childhood obesity. The objective of our study was to evaluate the Healthy Lifestyles Initiative, which aimed to strengthen community capacity for policy, systems, and environmental approaches to healthy eating and active living among children and families.

**Intervention Approach:**

The Healthy Lifestyles Initiative was developed through a collaborative process and facilitated by community organizers at a local children’s hospital. The initiative supported 218 partners from 170 community organizations through training, action planning, coalition support, one-on-one support, and the dissemination of materials and sharing of resources.

**Evaluation Methods:**

Eighty initiative partners completed a brief online survey on implementation strategies engaged in, materials used, and policy, systems, and environmental activities implemented. In accordance with frameworks for implementation science, we assessed associations among the constructs by using linear regression to identify whether and which of the implementation strategies were associated with materials used and implementation of policy, systems, and environmental activities targeted by the initiative.

**Results:**

Each implementation strategy was engaged in by 30% to 35% of the 80 survey respondents. The most frequently used materials were educational handouts (76.3%) and posters (66.3%). The most frequently implemented activities were developing or continuing partnerships (57.5%) and reviewing organizational wellness policies (46.3%). Completing an action plan and the number of implementation strategies engaged in were positively associated with implementation of targeted activities (action plan, effect size = 0.82; number of strategies, effect size = 0.51) and materials use (action plan, effect size = 0.59; number of strategies, effect size = 0.52). Materials use was positively associated with implementation of targeted activities (effect size = 0.35).

**Implications for Public Health:**

Community-capacity–building efforts can be effective in supporting community organizations to engage in policy, systems, and environmental activities for healthy eating and active living. Multiple implementation strategies are likely needed, particularly strategies that involve a high level of engagement, such as training community organizations and working with them on structured action plans.

## Introduction

Childhood obesity is a major public health concern in the United States and the world. The number of obese children and adolescents (aged 2–19 y) in the United States reached 12.7 million by 2013–2014, approximately 17% of the overall youth population (1). Obesity contributes to health problems, including physical problems (eg, diabetes, heart disease) (2,3) and psychological problems (eg, depression, anxiety) (4,5) and can last into adulthood (6). Additionally, obesity disproportionately affects marginalized communities, including racial/ethnic minority populations and people with low socioeconomic status (1).

Policy, systems, and environmental approaches are recommended as evidence-based practices for preventing childhood obesity, and these approaches are increasingly prevalent (7–9). For example, several national initiatives exist to increase community capacity for policy, systems, and environmental approaches to childhood obesity prevention (10–14). Such initiatives often include strengthening community capacity and linkages across multiple sectors to support healthy eating and active living (15), key priorities for obesity prevention. It is important to evaluate these efforts to determine whether they result in the desired outcomes and inform the refinement of strategies to maximize impact.

Implementation science has emerged in part to increase uptake of evidence-based practices into practice. Implementation science frameworks such as that posited by Proctor and colleagues (16,17) define *implementation strategies* as approaches for supporting the adoption and implementation of evidence-based practices (eg, across the community). Successful implementation strategies are expected to lead to multiple favorable implementation outcomes, such as increased uptake, penetration, fidelity, and sustainability of the practices in participating organizations (18). Although much of implementation science focuses on clinical service providers (19), community organizations can also be considered service providers, particularly through their role in providing supportive policy, systems, and environments for childhood obesity prevention.

We leveraged implementation science concepts to inform the evaluation of the Healthy Lifestyles Initiative, a multisector capacity-building initiative to support policy, systems, and environmental approaches to increase healthy eating and active living and reduce rates of childhood obesity and related disparities in the Kansas City, Missouri, and Kansas City, Kansas, metropolitan area. The Healthy Lifestyles Initiative sought to increase uptake and penetration of these approaches in participating organizations through the use of multiple implementation (ie, engagement) strategies. Rather than promoting adoption of a single evidence-based intervention, the Healthy Lifestyles Initiative promoted evidence-based policy, systems, and environmental practices (7–9) more generally to support adoption and implementation across a range of community sectors, including schools, child care providers, health care providers, businesses, nonprofit community organizations, and government organizations (eg, health departments, parks and recreation departments). Our evaluation, performed through a research–practice partnership, attempted to gauge the effect of the implementation strategies on uptake and penetration of the policy, systems, and environmental approaches targeted. Knowing whether and which implementation strategies used were effective can inform program refinements and continuation and support similar programs in other communities.

## Purpose and Objectives of the Healthy Lifestyles Initiative

A team of local stakeholders from the community and health care sectors, including Children’s Mercy Kansas City, participated in the 2011–2013 national learning collaborative, *Collaborate for Healthy Weight*, coordinated by the National Initiative for Children’s Healthcare Quality and supported by the Prevention and Public Health Fund (20–22). The team included representatives from the hospital’s community program and primary care clinics, the local health department, the YMCA, and a nonprofit community organization specializing in healthy lifestyle policy change. The collaborative supported nearly 40 teams in the United States in strengthening multisector linkages and driving policy and environmental improvements for healthy eating and active living in their local communities (23).

The Kansas City team’s participation in the collaborative resulted in the initiation of the Healthy Lifestyles Initiative in late 2013, led by members of Children’s Mercy Kansas City (24). Primary goals of the initiative were to promote 5 core implementation strategies to increase uptake and penetration of coordinated policy, systems, and environmental activities for childhood obesity prevention among community organizations and disseminate a consistent message on family healthy lifestyles. The Healthy Lifestyles Initiative conceptual model was mapped to Proctor and colleagues’ implementation science framework (Figure) (16).

**Figure:**
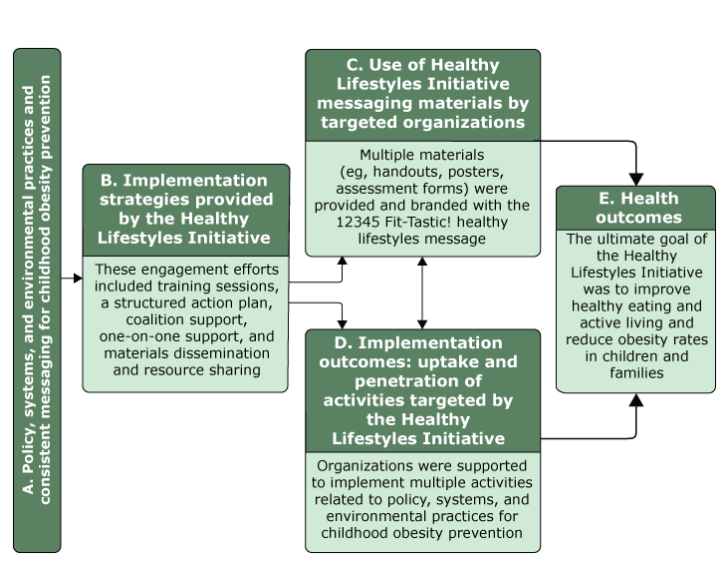
Conceptual model of the Healthy Lifestyles Initiative, Kansas City, Missouri, and Kansas City, Kansas, 2016. Model was mapped to Proctor and colleagues’ implementation science framework (16). Health outcomes were not assessed in this study.

## Intervention Approach

The key elements of the intervention were selected by using the framework provided by *Collaborate for Healthy Weight* (23), and the targeted health behaviors were informed through evidence review (12,25). The targeted outputs and activities of the initiative were for partners to implement policy, systems, and environmental activities (ie, implementation outcomes) in their organizations and in the communities in which they worked and incorporate the 12345 Fit-Tastic! message throughout their activities. The types of policy, systems, and environmental activities were broad, but activities generally were in these 8 categories: adopt new policy or change exiting policy, adopt new practices, create customized plans or goals with those served, develop or continue partnerships, initiate staff wellness activities, provide healthy lifestyles screenings or assessments, refer people served to primary care or other resources, and review organizational wellness policies.

A messaging campaign was developed to promote the targeted health behaviors (24,25). A public relations firm, working pro bono, presented multiple messaging options. Focus groups were conducted and stakeholders were polled to select the messages that resonated best. The message selected, 12345 Fit-Tastic!, promotes the following desired behaviors: 1 hour or more of physical activity, 2 hours maximum of screen time, 3 servings of low-fat or nonfat milk or yogurt, 4 servings of water (not sugary drinks), and 5 servings or more of fruits and vegetables per day. Extensive messaging materials and resources were developed and made available to partners (with their organization’s name and logo, if desired). All messaging materials and resources were made available on a website (https://fittastic.org) to all partners.

The Healthy Lifestyles Initiative used 5 implementation strategies to support organizations in using the messaging materials and implementing policy, systems, and environmental activities: 1) educational training, 2) a structured action plan, 3) coalition support, 4) one-on-one support, and 5) materials dissemination and resource sharing. These 5 strategies were mapped to the implementation strategies developed by Powell et al (26) (Table 1). Some strategies overlapped. For example, materials dissemination and resource sharing often took place during one-on-one support, and coalition support often involved educational training and one-on-one support.

**Table 1:** Description of Implementation Strategies Provided by the Healthy Lifestyles Initiative, Kansas City, Missouri, and Kansas City, Kansas, 2016

Implementation Strategy	Description	Delivery	Related Constructs From Powell et al (27)
Educational training	Training sessions included data on childhood obesity, background on the Healthy Lifestyles Initiative, and information about action planning strategies. Trainees also learned how to join the Healthy Lifestyles Initiative and how to gain access to resources, such as 12345 Fit-Tastic! messaging and educational materials.	Some training sessions were delivered to coalitions or collaborative groups. Other training sessions were provided to an organization’s staff members or other individuals. Most were provided upon request of the Healthy Lifestyles Initiative partner or through a grant or other project. Training sessions were also available on a quarterly basis after an open and ongoing regional stakeholder meeting.	Conduct educational meetings
Structured action plan	The action plan elements (“MAPPS for Change”) were based on elements presented to the National Institute for Children’s Health Quality’s initiative, Collaborate for a Healthy Weight: **M** —consistent use of **message** (12345 Fit-Tastic!); **A** — consistent **assessment** of weight status and lifestyle behaviors; **P** — customized healthy lifestyle **plan** for all; **P —** **policies and practices** that enable healthy eating and active living; and **S** — **statistics** or story telling about message reach, assessment/plans completed, and policy/environmental changes made.	All partners were provided information about the MAPPS for Change action plan when they signed up to join the Healthy Lifestyles Initiative. Training sessions and one-on-one support also included information about MAPPS for Change action planning. Partners could complete the MAPPS for Change action plan document through their partner account created on the initiative’s website. Paper copies of the MAPPS for Change action plan were provided at training sessions. All partners received 1) a reminder to complete their action plan when they logged into their partner account and 2) an annual email reminder to complete or update their plans.	Develop a formal implementation blueprint and obtain formal commitments
Coalition support	Supporting existing coalitions focused on healthy eating and/or active living was seen as a way to improve reach and impact of the intervention. This strategy was similar to a train-the-trainer approach, with a large amount of support being provided to the coalition leaders to infuse the Healthy Lifestyles Initiative framework throughout the coalition’s efforts.	Community coalition support included training coalition members (ie, staff from multiple organizations) and one-on-one contact with coalition leaders, primarily provided to 3 large coalitions on an ongoing basis. This support allowed for the coalition and its partners to have access to additional support and resources from the Healthy Lifestyles Initiative.	Promote network weaving and use train-the-trainer strategies.
One-on-one support	One-on-one support consisted of meetings or telephone or email support with Healthy Lifestyles Initiative partners beyond initial contact and/or outside of training. Most one-on-one support focused on generating or reviewing the MAPPS for Change action plan elements and providing assistance or guidance on these plans specific to the partner organization. One-on-one support also included linking together partners with similar agendas.	One-on-one support was offered to all partners when they joined the Healthy Lifestyles Initiative and was provided on request (in response to the initial contact or at a later date). This support sometimes occurred before an organization officially joining as a partner but more often occurred with established members to assist with the MAPPS for Change action plan, ideas for implementing action plan strategies, and partnerships.	Facilitation, provide local technical assistance, and provide ongoing consultation.
Materials dissemination and resource sharing	Materials dissemination and resource sharing most commonly included connecting Healthy Lifestyles Initiative partners with 12345 Fit-Tastic! educational and marketing materials. Also included were connection to other local and national resources (eg, resource guides, toolkits) and provision of data on childhood obesity or other information.	The 12345 Fit-Tastic! educational and marketing materials were available to all partners via the initiative’s website. Other information and resources were shared as requested by partners. At least quarterly, Healthy Lifestyles Initiative partners would receive email newsletters highlighting resources, partner stories, or new 12345 Fit-Tastic! materials.	Develop and distribute educational materials.

Potential partners (ie, recipients of the intervention) in various sectors — the community (eg, nonprofit organizations, fitness centers, grocery stores), education (eg, schools, child care), health care (eg, primary care, hospitals), and government (eg, health departments, parks and recreation) — that had programs addressing childhood obesity were identified through surveys, a comprehensive database of nonprofit organizations in the region, and networking. Organizations were invited to become a partner in the initiative through electronic newsletters, word of mouth, direct contact with existing networks (eg, state health agency; Special Supplemental Nutrition Program for Women, Infants, and Children [WIC] agencies; local health departments), and presentations at local, state, and regional conferences. Additional tactics were used to engage partners (eg, local health departments, WIC agencies) in underserved areas to address health disparities. These tactics included promotion through the social networks of selected partners and involvement and support from community leaders, such as the mayor of Kansas City, Kansas. By January 2016, the Healthy Lifestyles Initiative supported 218 community partners from organizations across Kansas and Missouri, primarily in the Kansas City metropolitan area.

## Evaluation Methods

The evaluation was conducted by research partners at Children’s Mercy Kansas City in 2016. Although implementation science was not used to inform the development of the intervention, the research–practice partnership used implementation science in the evaluation. The implementation strategies being delivered and implementation activities targeted by the Healthy Lifestyles Initiative were determined by the evaluators, and the effectiveness of the implementation strategies on uptake and penetration of the implementation activities targeted were evaluated by examining data collected through a brief online survey.

Any person who had signed up as a partner on the Healthy Lifestyles Initiative website before January 31, 2016, was eligible to participate in the survey. Multiple partners from a single organization could sign up and most often represented a unique program, effort, or location in their organization. Of the 218 partners representing 170 organizations, 144 (66%) had a moderate-to-high level of engagement with the intervention; assessment of engagement was based on whether the partner corresponded with intervention staff members after signing up on the website; 74 (34%) partners had no contact after signing up. The brief online survey was emailed to the 218 partners in early 2016 through REDCap, a secure web application for building and managing online surveys and databases (27). Self-report was selected as the measurement mode because of its appropriateness for capturing data on policy, systems, and environmental changes, and because objective measures were not feasible given the heterogeneity in types of policy, systems, and environmental activities targeted by partner organizations. Power analysis indicated that a moderate association (*r* = 0.3; effect size = 0.6) (28) between the implementation strategies and activities and outcomes implemented would be detectible with approximately 84 respondents, and a small-to-moderate association (*r* = 0.2; effect size = 0.4) detectible with 193 respondents. Complete responses were obtained from 80 partners, so this study was powered to detect moderate associations. This study was approved by the Children’s Mercy Kansas City human subjects protection committee.

### Measures

The survey assessed partners’ organization type, the implementation strategies engaged in, materials used, and activities implemented.

**Organization type.** Participants could select 1 of 6 organization types: child care provider, faith-based organization, health care provider, health department, school, and other. Responses in the “other” classification were then categorized, according to an open-ended response, as academic institution, business, nonprofit community organization, other government organization, and parks and recreation department.

**Implementation strategies engaged in**. Survey respondents identified the implementation strategies in which they had engaged (yes or no) from a list of 5 options: attended at least 1 Healthy Lifestyles Initiative training session; completed a Healthy Lifestyles Initiative action plan; participated in a community coalition supported by the Healthy Lifestyles Initiative; received one-on-one support from a Healthy Lifestyles Initiative staff member; and received materials and resources from a Healthy Lifestyles Initiative staff member. We summed the number of strategies each partner engaged in (0 to 5) and calculated a mean for all survey respondents.

**Materials used**. Partners reported the number of types of materials they used from a list of 10 Healthy Lifestyles Initiative materials: 1) assessment forms, 2) behavior trackers, 3) educational handouts, 4) flags or banners, 5) logos or graphics, 6) message cards, 7) newsletters, 8) posters, 9) social media, and 10) website. Partners were asked how frequently they used the materials: never (score, 0); 1 or 2 occasions (score, 1); 3 or 4 occasions (score, 2); monthly (score, 3); weekly (score, 4); and daily (score, 5). Data on the extent of material use were captured by an open-ended question that asked participants how, where, and to whom they had distributed or shared materials and an open-ended question on the extent to which the materials had been incorporated into their organization’s regular practice. Responses to the 2 items were combined and rated for each respondent on a scale of 0 to 5, with 5 indicating the most extensive use of materials. Ratings were coded independently by one research staff member and reviewed by another research staff member for agreement, and disagreements were reconciled. A materials index was developed for the 3 variables (number of types of materials used, frequency of use, and extent of use) to indicate overall level of use. The index was computed by standardizing each variable to a range of 0 to 100 (multiplying the variables by 10, 20, and 20, respectively). A mean index was calculated for all survey respondents. Higher scores indicate greater use of materials.

**Policy, systems, and environmental activities implemented**. Partners were asked, “Which of the following things have you done to support children’s healthy lifestyles?” A list of 8 implementation activities targeted by the Healthy Lifestyles Initiative was provided: 1) adopted new policy or changed existing policy, 2) adopted new practices, 3) created customized plans or goals with people served, 4) developed or continued partnerships, 5) initiated staff wellness activities, 6) provided healthy lifestyles screenings or assessments, 7) referred those served to primary care or other resources, and 8) reviewed organizational wellness policies. Participants also responded to an open-ended question about the extent of the activities implemented: how often they were engaging in the activities, whether activities were being incorporated into regular practice, and how many people were reached. Responses were rated on a scale of 0 to 5, with 5 indicating the most extensive implementation. Ratings were coded independently by one research staff member and reviewed by another research staff member, and disagreements were reconciled. An implementation activities index was developed from the 2 variables (number of activities implemented and extent of implementation) to indicate overall level of implementation. The index was computed by standardizing each variable to a range of 0 to 100 (multiplying the variables by 12.5 and 20, respectively) and then calculating a mean of the 2 variables. Higher scores indicate greater levels of implementation.

**Additional support needed**. An open-ended question was used to assess the types of additional supports partners desired from the Healthy Lifestyles Initiative. Responses were coded by inductive thematic analyses (29) by one coder and confirmed by another, and no discrepancies were found.

### Analysis

Means, standard deviations, and frequencies were computed for all study variables. To investigate overlap among the strategies and activities, Spearman correlations were assessed among implementation strategies engaged in and activities implemented. To investigate relationships of implementation strategies engaged in with materials used and activities implemented, the materials index and the implementation activities index were regressed in separate single-variable linear regression models on 1) each of the 5 implementation strategies engaged in and 2) the total number of implementation strategies engaged in. To investigate whether greater use of materials was related to a greater level of implementation, the implementation activities index (dependent variable) was regressed on the materials index. All independent variables were entered in separate models because of concerns about collinearity. For the 5 binary (yes or no) implementation strategies, an effect size was calculated by dividing the regression coefficient by the standard deviation of the dependent variable (28). A similar approach was used to calculate effect size for continuous independent variables, except the regression coefficient was first multiplied by the standard deviation of the independent variable. *P* < .05 was used to determine significance. All data were analyzed by using SPSS version 22.0 (IBM Corp).

## Results

Of the 218 surveys sent, 12 were returned because of an invalid email address, 126 had no response, and 80 were completed (39% response rate [80 of 206]). The 80 respondents represented 72 organizations and a mix of organization types; health departments, schools, health care providers, and nonprofit community organizations had the largest representation (Table 2). A larger proportion of survey respondents (65 of 80; 81%) had a moderate-to-high level of engagement with the intervention than did partners (144 of 218; 66%).

**Table 2:** Types of Organizations That Participated in the Healthy Lifestyles Initiative and Evaluation Study, Kansas City, Missouri, and Kansas City, Kansas, 2016^a^

Type	No. (%) of Organizations (n = 218)	Number (%) of Survey Respondents (n = 80)
Academic institution	3 (1.4)	2 (2.5)
Business	14 (6.4)	3 (3.8)
Child care provider	25 (11.5)	9 (11.3)
Nonprofit community organization	38 (17.4)	11 (13.8)
Faith-based organization	8 (3.7)	3 (3.8)
Health care provider	25 (11.5)	14 (17.5)
Health department	54 (24.8)	21 (26.3)
Other government organization	1 (0.5)	1 (1.3)
Parks and recreation department	8 (3.7)	1 (1.3)
School	42 (19.3)	15 (18.8)

a A brief online survey was emailed to 218 partners (defined as a person who signed up as a partner on the Healthy Lifestyles Initiative website before January 31, 2016) representing 170 organizations. More than 1 partner could represent a single organization. All data were self-reported.

Each Healthy Lifestyles Initiative implementation strategy was engaged in by 30.0% to 35.0% of survey respondents (Table 3). The median number of implementation strategies engaged in was 2.0 (interquartile range [IQR], 0–3.0). Correlations among the implementation strategies ranged from 0.07 (completing an action plan and receiving materials and resources) and 0.32 (receiving one-on-one support and receiving materials and resources).

**Table 3:** Descriptive Statistics for Healthy Lifestyles Initiative Implementation Strategies Engaged In, 12345 Fit-Tastic! Materials Used, and Activities Implemented by Participating Organizations in Kansas City, Missouri, and Kansas City, Kansas, 2016^a^

Category	No. of Respondents (%)^b^ (n = 80)
**Implementation strategies engaged in**	
Attended ≥1 training session	28 (35.0)
Completed an action plan	24 (30.0)
Participated in a community coalition	28 (35.0)
Received one-on-one support	26 (32.5)
Received materials and resources	26 (32.5)
No. of implementation strategies engaged in, median (IQR)^c^	2.0 (0–3.0)
**Materials used**	
Assessment forms	25 (31.3)
Behavior trackers	5 (6.3)
Educational handouts	61 (76.3)
Flags or banners	20 (25.0)
Logos or graphics	27 (33.8)
Message cards	23 (28.7)
Newsletters	13 (16.3)
Posters	53 (66.3)
Social media	16 (20.0)
Website	46 (57.5)
Number of different materials used^d^, median (IQR)	3.0 (2.0–5.0)
Frequency of materials used^e^, mean (SD)	3.3 (1.4)
Extent of materials use^f^, mean (SD)	2.5 (1.4)
Materials index^g^, mean (SD)	49.3 (21.7)
**Activities implemented**	
Adopted new policy or changed existing policy	18 (22.5)
Adopted new practices	23 (28.7)
Created customized plans or goals with people served	21 (26.3)
Developed or continued partnerships	46 (57.5)
Initiated staff wellness activities	29 (36.3)
Provided healthy lifestyles screenings or assessments	27 (33.8)
Referred those served to primary care or other resources	26 (32.5)
Reviewed organizational wellness policies	37 (46.3)
No. of different activities implemented^h^, mean (SD)	2.8 (2.2)
Extent of activities implemented^i^, median (IQR)	2.5 (1.0–4.0)
Implementation activities index^j^, median (IQR)	38.8 (22.5–61.2)

Abbreviations: IQR, interquartile range; SD, standard deviation.

a A brief online survey was emailed to 218 partners (defined as a person who signed up as a partner on the Healthy Lifestyles Initiative website before January 31, 2016) representing 170 organizations. More than 1 partner could represent a single organization. All data were self-reported.

b Unless otherwise indicated.

c Survey respondents identified the implementation strategies in which they had engaged from a list of 5 options. The number of strategies engaged in was summed for each respondent (range, 0–5).

d Survey respondents reported the number of types of materials they used from a list of 10 Healthy Lifestyles Initiative materials. The number of strategies engaged in (range, 0–10) was summed for each respondent.

e Survey respondents were asked how frequently they used the materials on a scale of 0 (never) to 5 (daily).

f Responses to 2 open-ended questions were rated on a scale of 0 to 5, with 5 indicating the most extensive use of materials.

g A materials index, scaled from 0 to 100, with 100 indicating greatest overall use, was developed from 3 variables (number of types of materials used, frequency of use, and extent of use) to measure overall use of materials.

h A list of 8 implementation activities targeted by the Healthy Lifestyles Initiative was provided. The number of strategies engaged in was summed for each respondent (range, 0–­8).

i Responses to 1 open-ended question were rated on a scale of 0 to 5, with 5 indicating the most extensive implementation.

j An implementation activities index, scaled from 0 to 100, with 100 indicating greatest level of implementation, was developed from 2 variables (number of activities implemented and extent of implementation) to indicate overall level of implementation.

Respondents used a median 3.0 (IQR, 2.0–5.0) of the 10 types of 12345 Fit-Tastic! materials. Educational handouts and posters were the most frequently used materials, by 76.3% and 66.3% of respondents, respectively. On average, the materials were used monthly. The mean materials index was 49.3 (standard deviation [SD], 21.7). More extensive material use included providing regular presentations on the Fit-Tastic! message, creating recipes and food samples or activity plans that aligned with the message, using components of the Fit-Tastic! message to promote weight-management goals for clients, and hanging Fit-Tastic! materials in an area likely to affect behavior (eg, cafeteria, grocery store produce section).

Respondents implemented a median of 2.5 (IQR, 1.0–4.0) of the 8 activities targeted by the Healthy Lifestyles Initiative. Developing or continuing community partnerships (57.5% of respondents) and reviewing organizational wellness policies (46.3% of respondents) were the most commonly reported implementation activities. The median implementation activities index was 38.8 (IQR, 22.5–61.2). Correlations among the implementation activities ranged from −0.10 (reviewed organizational wellness policies and created customized plans or goals with people served) and 0.58 (adopted new policy or changed existing policy and adopted new practices). Implementation activities that were more extensively used than others included incorporating physical activities and healthy food options (eg, walk breaks, adding healthy menu items, adding dance classes), improving nutritional quality of children’s food options at school, educating parents on healthy meal preparation, and adding healthy lifestyles screenings to primary care visits.

Two implementation strategies, attending training sessions (B = 10.41; *P* = .04; effect size = 0.48) and completing an action plan (B = 12.85; *P* = .01; effect size = 0.59), were associated with the materials index (Table 4). The total number of implementation strategies engaged in was also associated positively with materials used, with each additional implementation strategy related to a 5.65 higher score on the materials index (effect size = 0.52).

**Table 4:** Associations Among Healthy Lifestyles Initiative Implementation Strategies Engaged In, 12345 Fit-Tastic! Materials Used, and Activities Implemented in Kansas City, Missouri, and Kansas City, Kansas, 2016^a^

Variable	Materials Index^b^		Implementation Activities Index^c^	
	B (Standard Error)	*P* Value^d^	B (Standard Error)	*P* Value^d^
**Implementation strategies engaged in^e^**				
Attended ≥1 training session	10.41 (4.98)	.04	0.16 (6.12)	.98
Completed action plan	12.85 (5.11)	.01	22.70 (6.29)	.001
Received coalition support	1.21 (5.14)	.81	10.98 (6.32)	.09
Received one-on-one support	1.55 (5.29)	.77	−4.71 (6.51)	.47
Received materials or resources	4.31 (5.22)	.41	8.41 (6.42)	.19
Total implementation strategies engaged in^f^	5.65 (1.61)	.001	6.93 (2.06)	.001
**Materials used**				
Materials index^b^	—	—	0.45 (0.14)	.001

Abbreviation: B, unstandardized regression coefficient.

a A brief online survey was emailed to 218 partners (defined as a person who signed up as a partner on the Healthy Lifestyles Initiative website before January 31, 2016) representing 170 organizations. More than 1 partner could represent a single organization. All data were self-reported.

b A materials index, scaled from 0 to 100, with 100 indicating greatest overall use, was developed from 3 variables (number of types of materials used, frequency of use, and extent of use) to measure overall use of materials.

c An implementation activities index, scaled from 0 to 100, with 100 indicating greatest level of implementation, was developed from 2 variables (number of activities implemented and extent of implementation) to indicate overall level of implementation. A list of 8 implementation activities targeted by the Healthy Lifestyles Initiative was provided.

d Estimated by using linear regression.

e Survey respondents identified the implementation strategies in which they had engaged from a list of 5 options.

f The number of strategies engaged in was summed for each respondent (range, 0–5).

One implementation strategy, completing an action plan, was associated with the implementation activities index (B = 22.70; *P* = .001; effect size = 0.82). Receiving coalition support was associated with the implementation activities index (B = 10.98; *P* = .09; effect size = 0.40), although not significantly. The total number of implementation strategies engaged in was also associated positively with the implementation activities index, with each additional implementation strategy related to a 6.93 higher score on the implementation activities index (effect size = 0.51). The materials index was associated positively with the implementation activities index (effect size = 0.35).

Themes emerged on additional supports desired. Of the 45 partners who responded to this question, 19 (42%) desired more support for implementation, such as greater assistance with implementation planning and affecting policy change, and guidance in how to use and adapt the materials into their program or organization. Ten (22%) respondents desired different or additional materials, 8 (18%) desired additional training, and 8 (18%) desired increased communication with a designated contact person.

## Implications for Public Health

This study indicates that community-capacity–building efforts can increase uptake and penetration of policy, systems, and environmental approaches by community organizations for improving healthy eating and active living among children and families. In particular, partners who engaged in more Healthy Lifestyles Initiative implementation strategies engaged in more extensive implementation of policy, systems, and environmental activities. Completing an action plan was the most important implementation strategy. These findings suggest that community-capacity–building efforts, such as those led by the Centers for Disease Control and Prevention and local hospitals and health departments across the United States, have promise for improving policies, systems, and environments to prevent childhood obesity (7,9–13). Although evaluation of health and behavioral outcomes among children and families in these types of large-scale community-wide capacity efforts are often not feasible, implementation science frameworks (16) provide a useful model for gauging effects on implementation outcomes (ie, the intermediary factors that affect whether the efforts will ultimately improve health).

Partners who attended training sessions, completed an action plan, and engaged in more Healthy Lifestyles Initiative implementation strategies were more likely to use 12345 Fit-Tastic! materials. However, receiving materials or resources from the initiative team and receiving coalition support were not associated with use of these materials. The training sessions and action plan involved a higher level of engagement than other implementation strategies, suggesting that this higher level of engagement may be needed to support organizations to use healthy messaging materials. Many respondents reported the desire for more guidance on how to adapt the materials into their organization or program, and training sessions and action plans allow program staff members to provide such support. The action plan was often tailored to an organization; this type of tailoring may be needed, particularly by organizations that have less experience in supporting healthy eating and active living.

Completing an action plan was the single most important implementation strategy. About 30% of respondents completed an action plan, which is similar to the proportion completing each other strategy but is still a minority of participants. The action plan is a 1-page document that guides the sharing of a consistent message, implementation of a consistent assessment of weight status and healthy lifestyle behaviors and healthy lifestyles plan, and creation of policy or environmental changes to support healthy eating and active living. Organizations complete each section of the action plan with guidance from Healthy Lifestyles Initiative staff members, which may provide some accountability. Next steps in this research area should be to identify strategies for increasing the number of organizations that complete an action plan, which could be informed by investigating differences in organizational characteristics and implementation contextual factors (30) between completers and noncompleters. Organizations that used the healthy messaging materials were also more likely to engage in implementation activities, but the effect was smaller for using materials than for completing an action plan. Thus, disseminating messaging materials may increase organizations’ engagement in childhood obesity prevention, but messaging materials alone are not likely to be strong drivers of organization or behavior change and should be paired with policy, systems, and environmental supports such as training and action planning.

Each of the 5 implementation strategies was engaged in at a similar rate — by 30% to 35% of organizations. Thus, no single strategy seemed more likely to reach community partners than another strategy, but implementation rates increased for every additional implementation strategy engaged in. Two materials were most often used: the educational handouts and posters. A benefit of adopting healthy messaging materials from coordinated efforts such as the Healthy Lifestyles Initiative is that the messaging is consistent across materials and across organizations that adopt the materials. Consistency in messaging can maximize comprehension, minimize confusion, and increase awareness of selected health behaviors and policy, systems, and environmental changes needed to support the behaviors (12). More than half of survey respondents reported developing or continuing partnerships related to childhood obesity prevention, which appeared to be the most consistent benefit of the Healthy Lifestyles Initiative. Many organizations reported adopting new policies or practices and implementing multiple activities, which were primary targets of the initiative.

About one-third of partners had no correspondence with intervention staff members after signing up online. Initiatives such as the Healthy Lifestyles Initiative will have the greatest impact if they are able to reach a large portion of the targeted population of partners; maintaining partner engagement is critical. However, because available resources may limit the ability to engage all partners at ideal levels, engagement efforts may need to prioritize re-engagement of unengaged partners over supporting engaged partners or cultivating new partners. To this end, the Healthy Lifestyles Initiative used a train-the-trainer model to support highly engaged coalitions with a high reach into the target population of partners to engage and re-engage partners that may have otherwise had low levels of engagement. The initiative also prioritized resources toward continued engagement of organizations perceived to have a high reach into the target population and high potential for continued involvement and organizations representing various community sectors to maximize representation of all organizations engaged in obesity prevention. Future research should focus on improving understanding of why some organizations have low levels of engagement in such initiatives and strategies for increasing engagement. Our anecdotal evidence suggests staff turnover at partner organizations and competing priorities (eg, other grant projects) may have contributed to low levels of engagement.

This study is among the first to investigate how community-capacity–building efforts can increase implementation of policy, systems, and environmental approaches and use of consistent messaging among community organizations. Limitations include the lack of objective data, potential response and selection bias, and inability to determine causality. Organizations that were more involved in the initiative may have been more likely to respond to the survey, and in some cases multiple respondents represented a single organization, so findings may not generalize to nonresponders. Although it appeared that some implementation strategies, such as completing an action plan, led organizations to implement more activities, organizations that implemented more activities may have been more likely to participate in the initiative and complete the action plan (ie, reverse causality). Future studies could use objective measures, such as policy reviews, environmental audits, and body composition measures and leverage pre–post assessments, control groups, or interrupted time series designs. Assessing organizational characteristics (eg, availability of resources) and interviewing key informants could help uncover why some organizations engaged in the initiative more than others. Finally, practitioners could leverage implementation science to systematically select and tailor implementation strategies; many other effective (and potentially more effective) strategies may exist for improving community capacity for implementing policy, systems, and environmental practices (26,31).

Policy, systems, and environmental approaches are essential for advancing the culture of healthy eating, active living, and childhood obesity prevention (15). Numerous initiatives exist to build community capacity in these areas. However, more research is needed to evaluate and maximize the effectiveness of these initiatives. This study used an implementation science framework to investigate whether and which implementation strategies delivered by the Healthy Lifestyles Initiative were related to uptake and penetration of evidence-based practices. Findings suggest that community-capacity–building efforts can support community organizations in implementing policy, systems, and environmental activities but that multiple implementation strategies are likely needed, particularly activities that require a relatively high level of engagement, such as educational training and creating action plans.
